# Mitochondrial DNA Evidence for a Diversified Origin of Workers Building Mausoleum for First Emperor of China

**DOI:** 10.1371/journal.pone.0003275

**Published:** 2008-10-01

**Authors:** Zhi Xu, Fan Zhang, Bosong Xu, Jingze Tan, Shilin Li, Chunxiang Li, Hui Zhou, Hong Zhu, Jun Zhang, Qingbo Duan, Li Jin

**Affiliations:** 1 Ministry of Education (MOE) Key Laboratory of Contemporary Anthropology and Center for Evolutionary Biology, School of Life Sciences and Institutes of Biomedical Sciences, Fudan University, Shanghai, China; 2 Ancient DNA Laboratory, Research Center for Chinese Frontier Archaeology, Jilin University, Changchun, China; 3 Institute of Archaeology, Chinese Academy of Social Sciences, Beijing, China; 4 Shaanxi Provincial Institute of Cultural Relics and Archaeology, Xian, China; 5 Chinese Academy of Sciences and Max Planck Society (CAS-MPG) Partner Institute for Computational Biology, Shanghai Institutes for Biological Sciences, Chinese Academy of Sciences, Shanghai, China; University of Glasgow, United Kingdom

## Abstract

Variant studies on ancient DNA have attempted to reveal individual origin. Here, based on cloning sequencing and polymerase chain reaction-restriction fragment length polymorphisms, we analyzed polymorphisms in the first hypervariable region and coding regions of mitochondrial DNA of 19 human bone remains which were excavated from a tomb near the Terra Cotta Warriors and dated some 2,200 years before present. With the aim of shedding light on origins of these samples who were supposed to be workers building the mausoleum for the First Emperor of China, we compared them with 2,164 mtDNA profiles from 32 contemporary Chinese populations at both population and individual levels. Our results showed that mausoleum-building workers may be derived from very diverse sources of origin.

## Introduction

Ancient DNA methodology has made it possible for retrieving genetic information from historic and prehistoric material [1]. Variant studies on ancient DNA have attempted to uncover population history [2], [3], [4], [5], [6], [7] or identify specimens [8], [9], [10], [11]. As one of the preferred markers for ancient DNA analysis [12], [13], mitochondria DNA (mtDNA) shows great advantage over nuclear DNA when dealing with fragmented, chemically modified, and trace amount of DNA from both historical and prehistorical specimens [14], [15], owing largely to its high copy number, rapid mutation rate, absence of recombination, and maternal inheritance.

Ying Zheng was the First Emperor of China, who ended the Warring States Period, established the first empire of China (Qin Dynasty) in 221 BC and died in 210 BC. According to historical records, it took 39 years and 720,000 workers to build an amazingly magnificent mausoleum (about 220,000 square meters, much larger than the pyramid of Khufu) for him. Since the population size of Qin Dynasty was twenty-two millions and it controlled a vast territory, the origin of these workers became an intriguing mystery. Between February and March 2003, 121 human skeletons were excavated by a team from Archaeology Institute of Shannxi when cleaning up a Qin-Dynasty kiln 500 meters away from the site where Terra Cotta Warriors were found. According to morphological observation, some bones were thickset with different extent of arthritis, and others bore fractures or obvious adaptation structures caused by intense pull from muscle, suggesting that these people were engaged in heavy work before death. Furthermore, given their casual and layer-on-layer burial accompanied by potsherds, iron tools, and several instruments of torture in a 10-meter deep tomb, they would have been of very low social status and logically supposed to be mausoleum-building workers (MBW) for the First Emperor of China. Clarification of their origins is imperative to further understanding of the Terra Cotta Warriors and the history of Qin Dynasty. Owing to poor conservation (e.g. eroding by flood), however, it was regrettably difficult to measure crania, leading to inability to identify their origins from physical morphology.

Aiming at uncovering MBWs' origin from genetic information, we investigated mitochondrial lineages of these bone specimens under the hypothesis that MBSs were brought in from various geographic areas.

## Materials and Methods

Overall 121 human thighbone specimens were taken from Archaeology Institute of Shannxi (China), and they were of different individuals according to physical examination. Since the quality of the samples is similar by visual inspection, 50 specimens were randomly chosen for the following analysis.

### Extraction, DNA amplification and sequencing (Fudan University, Shanghai, China)

To eliminate contamination from prior handling, the outer layer of bone was removed, then soaked in bleach (∼5% sodium hypochlorite solution) for 15 min [16], [17], rinsed in ethanol (70%), and followed by 30 min UV-irradiation for each face. Then each bone was cut into small fragments and ground into a fine power in SPEX SamplePrep Freezer/Mills 6750. Subsequently, about 500 mg bone powder was used for a silica-base DNA extraction as described [18]. At least two independent samplings and extractions were obtained from each remain.

Thermal cycler conditions consisted of initial 15 min incubation at 94°C followed by 45 cycles of 30 s at 94°C, 30 s at 52°C, and 30 s at 72°C, with a final extension step at 72°C for 7 min using three pairs of overlapping primers L16053 (5′-GGGAAGCAGATTTGGGTAC-3′), H16158 (5′-GATGTGGATTGGGTTTTTATG-3′); L16154 (5′-TACCATAAATACTTGACCACCTG-3′), H16265 (5′-GTTTGTTGGTATCCTAGTGGG-3′); L16263 (5′-AACTCCAAAGCCACCCC-3′), H16366 (5′-TGAGGGGGGTCATCCAT-3′); L16016 (5′- ATTCTCTGTTCTTTCATGGG-3′) and H16403 (5′- ATTGATTTCACGGAGGATGG -3′) (numbering according to the revised Cambridge reference sequence(rCRS)[19], Genbank accession number AC_000021). The 25 µl reaction mix contained 1U of *rTaq* polymerase (Takara), 200 µM of each dNTP, 1.5 mM Mg^2+^, 1 µM of each primer and 1 µg Bovine Serum Albumin (BSA, Takara) in order to eliminate some effects of the PCR inhibitors from both extraction and specimens [20].

Amplification products of the correct size were purified with AxyPrep PCR Clean-up Kit (Axygen) and subsequently sub-cloned using TA cloning kit (Takara) according to the manufacturer's instruction. Eight clones of each product, with inserts of the expected size identified by PCR using primers RV-M (5′-GAGCGGATAACAATTTCACACAGG-3′) and M13-47 (5′-CGCCAGGGTTTTCCCAGTCACGAC-3′), were purified by means of Shrimp Alkaline Phosphatase (SAP) and Exon I and afterwards sequenced with BigDye Terminator kit (Applied Biosystems) on an ABI 3130xl Genetic Analyzer (Applied Biosystems) using primers RV-M or M13-47.

### MtDNA assignment

By searching for haplogroup specific HVR I motif and matching with available modern data sets, the ancient mtDNAs obtained were tentatively assigned to hgs. In addition, hg diagnostic coding region polymorphisms were typed using PCR-RFLP (restriction fragment length polymorphism) [21]. PCR protocol was the same as above with primers listed in Table S1.

### Independent replication (Jilin University, Jilin, China)

The surface of each bone fragment was removed, soaked in bleach for 10 min, washed with 100% ethanol, UV-irritated for 30 min on each side and ground into powder with SPEX 5810. DNA was extracted from bone powder using GENECLEAN Kit® For Ancient DNA (Q-BIO gene) according to the manufacturer's instruction. HVR I fragment (16035-16398) of ancient mtDNA was amplified with two pairs of overlapping primers L16017 (5′-TTCTCTGTTCTTTCATGGGGA-3′), H16251 (5′-GGAGTTGCAGTTGATGTGTGA-3′), L16201 (5′-CAAGCAAGTACAGCAATCAAC-3′), and H16409 (5′-AGGATGGTGGTCAAGGGA-3′).

Thermal cycler conditions consisted of initial 4 min incubation at 94°C followed by 6 cycles of 1 min at 94°C, 55 s at 58°C, and 1 min at 72°C and 34 cycles of 1 min at 94°C, 55 s at 54°C, and 1 min at 72°C, with a final extension step at 72°C for 10 min. The 12.5 µl reaction mix contained 1 U of *Taq* polymerase, 200 µM of each dNTP, 2.5 mM MgCl_2_, 50 mM KCl, 2 µM of each primer and 2 mg/mL BSA. PCR products were directly sequenced with ABI 310 Terminator sequencing Kit (Applied Biosystems, Foster, USA) using the same primers as PCR.

### Authentication

Standard precaution and measures for ancient DNA analysis with object to demonstrate authenticity of the obtained data were strictly followed [1], [22], [23], [24]. All samples were excavated recently and had been kept in room temperature storage with minimal handling. This is important as previous studies found a high proportion of contaminants on museum materials [25] although the contamination level varies [26] presumably as a consequence of the extent of handling. All pre-PCR work was performed in a laboratory exclusively dedicated to ancient DNA manipulation and physically separated from the laboratory in which PCR cycling and post-PCR analysis was conducted. Additionally, a one-way (pre-PCR→PCR→post-PCR) procedure was always followed to avoid the imperceptible carrying of DNA aerosols on clothes or skin into the ancient laboratory [27]. Disposable tools, masks, gloves, laboratory coats, and filter-plugged tips were used and changed frequently to avoid cross-contamination. To detect possible contamination, negative controls were implemented for each sample for extraction and PCR. To trace possible contamination, mtDNA sequences from the authors and other laboratory members who had manipulated the bones were obtained. Only independent extractions and amplifications yielding identical sequences with all controls being negative were included in the subsequent analyses. Primers covering three overlapping fragments were used for reducing the likelihood that a nuclear insertion rather than the organelle mtDNA was amplified [23], [28], [29]. Since lesions in the ancient DNA template would be expected to be non-reproducible from different extracts [30] and artifacts at a given site caused by low fidelity of polymerase and sequencing error, as well as jumping PCR [31], [32], were detectable across clones, two independent extractions and cloning sequencing were conducted. Sequences were aligned and compared across clones. Hgs were validated by both HVR I motifs and hg specific coding region SNPs. Moreover, to identify potential laboratory-based contamination, independent replication was conducted at a separate laboratory (Ancient DNA Laboratory, Research Center for Chinese Frontier Archaeology, Jilin University) exclusively dedicated to ancient DNA manipulation. Nevertheless, for the confirmation that DNA was present in the sample, we relied on replicated extraction and amplification both within and between laboratories as done in various other studies [30], [33], [34] rather than amino acid racemization. To detect ‘long’ range amplification of ancient DNA [15], [22], [35], an alternative 408-bp PCR fragment using primers L16016 (5′- ATTCTCTGTTCTTTCATGGG-3′) and H16403 (5′- ATTGATTTCACGGAGGATGG-3′) were amplified.

### Sequence analysis

Overall 2,164 mtDNA profiles from previous published mtDNA data of 32 Chinese populations (Table S2, Figure S1) were taken as representatives of contemporary Chinese and used for comparison with MBWs. The 32 contemporary Chinese populations were grouped as Northern Han (NH), Southern Han (SH), Northern Minorities (NM) and Southern Minorities (SM) according to their geographic locations and ethnic affiliation and knowledge on significant differentiation between Northern and Southern Hans [36], [37]. Gene diversity and nucleotide diversity were calculated using ARLEQUIN version 3.1 [38]. Principal component (PC) plot were obtained using SPSS for Windows Release 11.0.1 (15^th^ Nov 2001). Median-joining networks of hgs were constructed using Network 4.5.0.0 (www.fluxus-engineering.com).

## Results

Among 50 bone specimens for which DNA was extracted, 19 yielded replicable profiles within laboratory, two (M54, M57) of which were sent for independent replication at Jilin University and yielded consistent results. These 19 authenticated samples (Table 1) were included in the subsequent analysis. The other 31 specimens include 22 with no amplifiable DNA and 9 failed to be replicated within laboratory.

**Table 1:** MtDNA HVR I motif and RFLP results of MBWs and individual haplogroup assignment.

Sample	Hg	HVR I Variation (-16000)	663	5176	9 bp	12406	13262	10400	10398	4833
			Hae III	Alu I		Hpa I	Alu I	Alu I	Dde I	Hha I
M7	M8a	184-189-192-223-298-311-319	-	+	2	+	-			
M17	B5b	140-183C-189-243-355	-	+	1	+	-			
M21	F*	207-304-311	-			-	-	-	-	
M31	N*	223-245		+				-	+	
M37	F1b	183C-189-195G-232A-249-258C-304-311				-		-	+	
M39	F1b	189-232A-249-265C-304-311	-			-		-	-	
M41	N9a	111-129-223-257A-261						-	-	
M43	N9a	129-223-257A-261	-	+	2	+	-			
M47	N9a	223-257A-261-311						-	+	
M50	M7a	209-223						+	+	
M53	D*	223-299-325-362	-	-	2	+	-			
M54**★**	C	223-239-298-327-357					+	+	+	
M57**★**	F1a1	129-145-162-172-304	-	+	2	-	-			
M60	G2a	92-102-223-227-272-278-319-362						+	+	+
M61	D5	164-172-182C-183C-189-223-362	-	-	2	+	-			
M71	F1a1	162-172-304	-			-		-	-	
M86	A	223-290-311-319-362	+	+	2	+	-			
M91	B5	140-183C-189-362	-	+	1	+	-			
M98	B4a	182C-183C-189-217-261-360	-	+	1	+	-			

NOTE. Specimens with **★** were independently replicated in Jilin University; Sites are numbered according to the revised CRS of Andrews et al. (1999). The suffixes A, G, and C indicate transversions; ‘-’ and ‘+’ denote the absence and presence of the restriction site, respectively. ‘1’ denotes the presence of the 9-bp (CCCCCTCTA) deletion, ‘2’ denotes nondeletion (i.e., two repeats of the 9-bp fragment).

**Figure 1:**
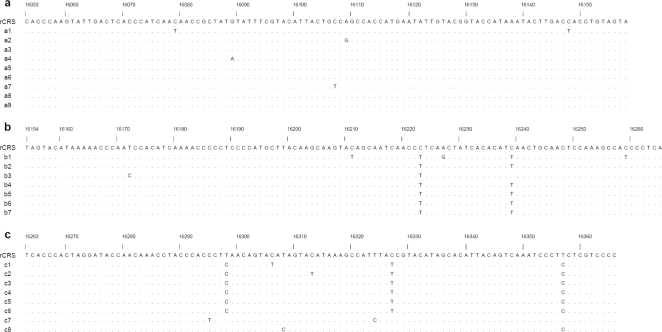
Alignment of cloned mtDNA sequences from sample M54. rCRS, revised Cambridge reference sequence [19]; A dot indicates identity with respect to rCRS; a, b and c are clones from the fragment between nucleotides (nt) 16053–16158, 16154–16265, and 16263–16366. The transition at nt 16223, 16239, 16298, 16327 and 16357 were observed in most of clones, and are presumed to be true transition in the authentic DNA. The diverging sequences (c7 and c8) are considered to represent exogenous DNA, but they do not match any of staffs (Table 3). The substitutions at nt 16079, 16089, 16107, 16109, 16148, 16172, 16211, 16227, 16259, 16306, 16313, are presumed to be the result of scattered miscoding lesions, which is a characteristic finding for ancient DNA [30].

All the 19 authenticated specimens showed appropriate ancient DNA molecular behaviors including chimeric sequences [39], [40] observed as difference among clones (Figure 1), and failure to amplify a 408-bp PCR fragment. No sequences showed obvious conflict with hg-defining segregating sites except for 16102 T->C in M60, which was a rare substitution found only in one published study [41]. A total of 314 nucleotides, corresponding to 16053–16366 in rCRS [19] were sequenced. The total alignment included 45 substitutions, of which 38 were affected by transitions and 7 by transversions, but no deletion and insertion were observed.

**Table 2:** Miscoding lesions observed within clones from MBWs.

	Transition				Transversion				
	Type 1		Type 2						
Sample	T->C	A->G	C->T	G->A	G->T	A->T	C->A	T->G	A->C
M7			4						
M17	3	1	2		2	1			
M21	1		5	1					1
M31	2	1	1	2					
M37	4		3						1
M39	4	4	1						
M41	3	3	7	1					
M43			4				1		
M47	3		3				1		
M50		1	4						
M53		2	4	1				1	
M54	2	2	7	1					
M57	1		2	2	1				
M60		3	5						
M61	1		1	1					
M71	1		4	1					
M86	4						1		1
M91	3		2				1		
M98	1					1			
Total	33	17	59	10	3	2	4	1	3

**Table 3:** Nucleotide substitutions and mtDNA haplogroups assignments for staffs involved in the excavation and analysis of MBWs.

Subject	HVR I region (-16000)	Haplogroup
F1	66 223 311	M*
F2	223 234 290 362	M*
F3	172 223 356 362	Not determined
F4	223 234 291 316 362	M9a
F5	189 223 311	M*
F6	111 129 140 183C 189 200T 243	B5b
A1	68 86 189 271 304 311	F1
A2	223 362	Not determined
J1	136-183C-189-217-218-239-248	B4b1
J1	126-174-223-311-362	Not determined
J1	145-182C-183C-189-217-261-360	B4a

F1–F6, staffs of the research laboratory at Fudan University; A1 and A2, archaeological and anthropological staffs; J1–J3, staffs of the research laboratory at Jilin University.

Variations out of the range 16053–16366 in the modern data set were excluded for analysis. The gene diversity and nucleotide diversity of mtDNA HVR I of MBWs was 1.000 (S.E. 0.017) and 0.019 (S.E. 0.010), respectively, which were both slightly higher than 32 extant populations (Table S3), despite non-significant differences (p>0.05).

**Figure 2:**
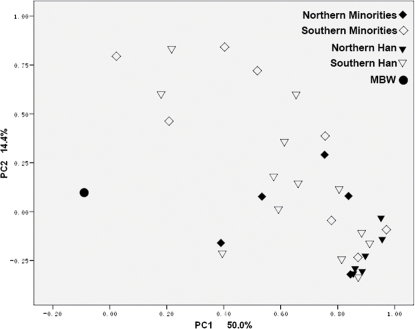
Principal component analysis of estimated percentage of shared haplogroups among MBWs and 32 Chinese populations. The 32 Chinese populations were divided into four groups (see Table S2).

To generate a PC plot (Figure 2), estimated percentage of hgs shared between MBWs and 32 modern populations were calculated (Table S3) and hgs absent in MBWs were excluded. The MBWs is an obvious outlier without showing any close affiliation with any of the extant populations.

The 19 specimens could be classified into 15 hgs, none of which that seem to be specific in the extant west Eurasian populations. The MP (Maximum parsimony) trees of median-joining networks of 13 hgs were constructed and shown in Figure S2 where MBWs were marked as solid circles. The network for hg M7a and B5 were not constructed due to data scarcity in the 2,164 mtDNA profiles. The clustering of each MBW profile with those observed in the extant populations may reveal the possible sources although cautions should be taken considering the fact that more recent migrations may have affected the hg frequencies in the extant populations. Nevertheless, the MBWs might be derived from very diverse sources. In particular, M86 (hg A) shared HVR1 variation with a NH from Qinghai Province. M98 (hg B4a) clustered with SM, indicating this worker might come from a minority of southern China. It should be noted that the frequency of hg B4a is high in SM [42]. M17 (hg B5b) was likely a SH on account of its position, and M60 (hg G2a) seemed likely to come from Han. M61 (hg D5) closely resembled with some SMs, and M21 (hg F*) clustered together with some NHs. Given high frequency of hg F1a1 in Southeast Asia [43], M57 and M71 (both with hg F1a1) might be originated from the south. Since hg D* was present with high frequency in both NM [44], [45] and SM [42] and rare in Han [46], M53 (hg D*) could be excluded from Han. However, MP trees are uninformative in revealing the sources of M37 (hg F1b), M39 (hg F1b), M41 (hg N9a), M43 (hg N9a), M47 (hg N9a), M31 (hg N*), M54 (hg C) and M7 (hg M8a). The network for hg M7a and B5 were not constructed due to the lack of sufficient data in our current database on Chinese, thus the affiliation of M91 (hg B5) and M50 (hg M7a) could not be inferred.

## Discussion

In the present study, we successfully extracted and analyzed maternal genetic information from 19 MBWs, the low amplification success (38%) as well as the difference of amplification between two sets of primers was in agreement with the poor conservation. However, the independent replication in Jilin University was carried out using primers targeting fragments longer than 200 bp and yielded consistent results, indicating that these 19 MBWs were in better conservation. Based on the high diversity of aDNAs (Table S3), the likelihood that they were from contaminants of several persons or cross-contamination was very low. Moreover, the failure of amplifying the 408-bp PCR fragment provided indirect evidence that longer templates were not present in large numbers. Miscoding lesions observed within clones of PCR products (Table 2, Figure 1) showed that Type 2 (cytosine->thymine/guanine->adenine) miscoding lesions represent the majority of damage-derived miscoding lesions in most of MBWs. This was consistent with previous studies [47], [48], though there were several transversions and more Type 1 (thymine->cytosine/adenine->guanine) miscoding lesions than Type 2 in some specimens, due to *Taq* polymerase error. Concerning the elaborate precaution we adopted and phylogenetic analysis, as well as the typical characteristic of ancient DNA, we believed that these 19 mtDNA HVR I sequences were authentic.

It is likely that MBWs was an admixture of East Asians. First, this 19-individual population was surprisingly more diverse than any of the 32 populations. In parallel, PC plot (Figure 2) of estimated percentage of shared hgs strongly supported that MBWs was obviously distinct from all 32 compared populations on first and second principal component. According to phylogeny of mtDNA lineages [46], [49], [50], these 19 specimens of MBWs could be assigned to 15 east-Eurasia specific hgs.

In particular, four specimens (M86, M60, M21 and M17), accounting for 21%, could be Han in origin, while three specimens (M98, M61 and M53, 16%) were likely from SM. Overall seven specimens (M17, M60, M21, M98, M61, M57 and M71, 37%) were likely originated from the south, while only one (M86, 5%) was from the north. According to a previous study of frequency distribution of dominating mtDNA hgs [51], M7 (hg M8a), M53 (hg D*), M54 (hg C), M60 (hg G2a), M61 (hg D5) and M86 (hg A), accounting for 32%, might come from the north, while M17 (hg B5b), M21 (hg F*), M31 (hg N*), M37 (hg F1b), M39 (hg F1b), M50 (hg M7a), M71 (hg F1a1), M57 (hg F1a1), M91 (hg B5) and M98 (B4a), accounting for 53%, were likely from the south. We therefore postulated that both Hans and minorities were recruited for building mausoleum, but many were from South China. This might, if not impossible, reflect the origin of 720,000 workers, provided that these 19 MBWs might be representative of them. Additionally, no specimen was distinctively from NM, and it did not come as a surprise because of geographic locations of NM, which are a little far away from the territory of Qin Dynasty as shown in Figure S1.

Interestingly, the specimen M50 (HVR I motif 16209–16223) belonging to hg M7a, had the same variation with a Japanese [52] and several Ryukyuans [53], [54]. Given that M7a has a much higher frequency in Ryukyuans [43] with the greatest Asian diversity (83%) than in Chinese [54], it seemed likely that this worker had a relatively closer genetic affinity with the ancestors of modern Japanese.

In conclusion, we showed that MBWs was an admixture and bore genetic continuity with contemporary Chinese populations. Its origin was much diversified, which seems to be compatible with historical accounts that the sources of slaved workers at Qin Dynasty tend to be extremely diverse. Furthermore, we showed that a strong presence of the workers of southern origins although the results of analysis should be taken with caution in the context of more recent migrations after Qin Dynasty. Further studies are important to provide a more definitive understanding on the origin of these samples using the whole genome of mtDNA and Y chromosomal variations.

### Electronic-Database Information

Accession numbers and the URL for data in this article are as follows: GenBank Overview, http://www.ncbi.nlm.nih.gov/Genbank/GenbankOverview.html (for mtDNA control region data; accession numbers EU700062-EU700080).

## Supporting Information

Table S1 Primers for mtDNA coding region SNPs assay with PCR-RFLP
(0.03 MB DOC)

Table S2 Overall 32 populations used for comparison
(0.13 MB DOC)

Table S3 Estimated percentages of haplogroups shared among MBWs and modern Chinese populations, as well as gene diversity of each population.
(0.11 MB DOC)

Figure S1 Geographic locations of the populations considered in this study.
(6.74 MB TIF)

Figure S2 Maximum Parsimony (MP) trees of median-joining networks
(9.69 MB TIF)
