# Supplementary material for: Mitochondrial DNA Evidence for a Diversified Origin of Workers Building Mausoleum for First Emperor of China
Source: PLoS One. 2008 Oct 1;3(10):e3275. doi: 10.1371/journal.pone.0003275 (PMC2557057; doi:10.1371/journal.pone.0003275)
Supplement: Table S1 — Primers for mtDNA coding region SNPs assay with PCR-RFLP (0.03 MB DOC) [file pone.0003275.s001.doc]

Table S1. Primers for mtDNA coding region SNPs assay with PCR-RFLP

| SNP Locus | Enzyme | Hg | Primers (5'-3') | Product Length |
| --- | --- | --- | --- | --- |
| 663 | Hae III | A | L629-CACATCACCCCATAAACAAAT H742-ATCGTGGTGATTTAGAGGGT | 113bp |
| 5176 | Alu I | D | L5135-CTCCAGCACCACGACCCTAC H5253-GCAAAAAGCCGGTTAGCG | 119bp |
| 12406 | Hpa I | F | L12362-CCCTAACCCTGACTTCCCTAAT H12449- AAGGTGGATGCGACAATGG | 106bp |
| 13262 | Alu I | C | L13188-CACTCTGTTCGCAGCAGTCTG H13280-GTTGGTTGATGCCGATTGTAA | 113bp |
| 9-bp |  | B | L8226-TTCCCCTAAAAATCTTTGAAATA H8325-AAAAGGTTAATGCTAAGTTAGCTTTA | 100 or 91bp |
| 4833 | Hha I | G | L4798-CCTTTCACTTCTGAGTCCCA  H4914- GTGAGGGAGAGATTTGGTATATG | 117bp |
| 10398 | Dde I | M/N | L10341- ATCATCATCCTAGCCCTAAGTC H10439- GTCGAAATCATTCGTTTTGTT | 99bp |
| 10400 | Alu I |
