# Supplementary material for: Mitochondrial DNA Evidence for a Diversified Origin of Workers Building Mausoleum for First Emperor of China
Source: PLoS One. 2008 Oct 1;3(10):e3275. doi: 10.1371/journal.pone.0003275 (PMC2557057; doi:10.1371/journal.pone.0003275)
Supplement: Table S2 — Overall 32 populations used for comparison (0.13 MB DOC) [file pone.0003275.s002.doc]

Table S2. Overall 32 populations used for comparison

|  | **population** | **code** | **size** | **Reference** |
| --- | --- | --- | --- | --- |
| Northern Han (NH) | Shannxi | 1 | 53 | [1] |
|  | Qinghai | 2 | 44 | [1] |
|  | Neimeng | 3 | 45 | [1] |
|  | Xian | 4 | 85 | [2] |
|  | Liaoning | 5 | 51 | [1] |
|  | Gansu | 6 | 45 | [1] |
| Southern Han (SH) | Zhejiang | 7 | 61 | [1] |
|  | Yunnan | 8 | 59 | [1] |
|  | Taiwan | 9 | 66 | [3] |
|  | Sichuan | 10 | 70 | [1] |
|  | Shanghai | 11 | 56 | [1] |
|  | Jiangxi | 12 | 23 | [1] |
|  | Jiangsu | 13 | 67 | [1] |
|  | Hunan | 14 | 16 | [1] |
|  | Guangxi | 15 | 29 | [1] |
|  | Fujian | 16 | 54 | [1] |
|  | Changsha | 17 | 82 | [2] |
|  | Anhui | 18 | 42 | [1] |
|  | Guangdong | 19 | 29 | [4] |
| Northern Minorities (NM) | Mogolian | 20 | 48 | [5] |
|  | Korean | 21 | 48 | [5] |
|  | Kazak | 22 | 53 | [6] |
|  | Ewenki | 23 | 47 | [5] |
|  | Tibetan | 24 | 56 | [7] |
|  | Uygur | 25 | 47 | [6] |
| Southern Minorities (SM) | Yi | 26 | 56 | [8] |
|  | Tujia | 27 | 96 | [7] |
|  | Yao | 28 | 395 | [8] |
|  | Miao | 29 | 142 | [8] |
|  | Dai | 30 | 38 | [4] |
|  | Bai | 31 | 55 | [7] |
|  | Zhuang | 32 | 83 | [4] |

1. Wen B, Li H, Lu D, Song X, Zhang F, et al. (2004) Genetic evidence supports demic diffusion of Han culture. Nature 431: 302-305.

2. Oota H, Kitano T, Jin F, Yuasa I, Wang L, et al. (2002) Extreme mtDNA homogeneity in continental Asian populations. Am J Phys Anthropol 118: 146-153.

3. Horai S, Murayama K, Hayasaka K, Matsubayashi S, Hattori Y, et al. (1996) mtDNA polymorphism in East Asian Populations, with special reference to the peopling of Japan. Am J Hum Genet 59: 579-590.

4. Yao YG, Nie L, Harpending H, Fu YX, Yuan ZG, et al. (2002) Genetic relationship of Chinese ethnic populations revealed by mtDNA sequence diversity. Am J Phys Anthropol 118: 63-76.

5. Kong QP, Yao YG, Liu M, Shen SP, Chen C, et al. (2003) Mitochondrial DNA sequence polymorphisms of five ethnic populations from northern China. Hum Genet 113: 391-405.

6. Yao YG, Kong QP, Wang CY, Zhu CL, Zhang YP (2004) Different matrilineal contributions to genetic structure of ethnic groups in the silk road region in china. Mol Biol Evol 21: 2265-2280.

7. Wen B, Xie X, Gao S, Li H, Shi H, et al. (2004) Analyses of genetic structure of Tibeto-Burman populations reveals sex-biased admixture in southern Tibeto-Burmans. Am J Hum Genet 74: 856-865.

8. Wen B, Li H, Gao S, Mao X, Gao Y, et al. (2005) Genetic structure of Hmong-Mien speaking populations in East Asia as revealed by mtDNA lineages. Mol Biol Evol 22: 725-734.
