# Supplementary material for: Mitochondrial DNA Evidence for a Diversified Origin of Workers Building Mausoleum for First Emperor of China
Source: PLoS One. 2008 Oct 1;3(10):e3275. doi: 10.1371/journal.pone.0003275 (PMC2557057; doi:10.1371/journal.pone.0003275)
Supplement: Table S3 — Estimated percentages of haplogroups shared among MBWs and modern Chinese populations, as well as gene diversity of each population. (0.11 MB DOC) [file pone.0003275.s003.doc]

Table S3. Estimated percentages of haplogroups shared among MBWs and modern Chinese populations, as well as gene diversity of each population. For each population’s code and references see Table S2.

|  |  |  | **Estimated percentage of mtDNA Haplogroup (%)** | | | | | | | | | | | | | | |  | Gene diversity (S.D.) |
| --- | --- | --- | --- | --- | --- | --- | --- | --- | --- | --- | --- | --- | --- | --- | --- | --- | --- | --- | --- |
| Group | Population | size | A | B4a | B5 | B5b | C | D* | D5 | F* | F1a1 | F1b | G2a | M7a | M8a | N* | N9a | others |  |
|  | MBWs | 19 | 5.26 | 5.26 | 5.26 | 5.26 | 5.26 | 5.26 | 5.26 | 5.26 | 10.53 | 10.53 | 5.26 | 5.26 | 5.26 | 5.26 | 15.79 |  | 1.000(0.017) |
| Northen  Han (NH) | 1 | 53 | 5.66 |  |  |  | 1.89 | 20.75 | 1.89 | 3.77 | 5.66 | 1.89 |  |  | 1.89 | 5.66 | 1.89 | 49.06 | 0.999(0.004) |
|  | 2 | 44 | 9.09 |  |  |  | 2.27 | 18.18 | 9.09 | 2.27 | 2.27 |  | 4.55 |  | 6.82 | 2.27 | 2.27 | 40.91 | 0.996(0.006) |
|  | 3 | 45 | 4.44 | 4.44 |  | 2.22 | 4.44 | 22.22 | 2.22 | 2.22 | 2.22 | 2.22 |  |  | 2.22 |  | 6.67 | 44.44 | 0.995(0.006) |
|  | 4 | 84 | 8.24 | 1.18 |  | 3.53 | 4.71 | 24.71 | 5.88 | 3.53 |  |  |  | 1.18 |  | 1.18 | 3.53 | 42.35 | 0.998(0.002) |
|  | 5 | 51 | 3.92 | 3.92 |  | 1.96 | 1.96 | 21.57 | 3.92 |  | 3.92 | 1.96 | 1.96 |  | 7.84 | 1.96 | 3.92 | 41.18 | 0.991(0.008) |
|  | 6 | 45 | 17.78 |  |  |  |  | 24.44 |  | 2.22 | 6.67 |  | 2.22 |  | 4.44 | 2.22 |  | 40 | 0.992(0.007) |
| Southern  Han (SH) | 7 | 61 | 4.92 | 6.56 | 1.64 | 1.64 | 6.56 | 6.56 | 1.64 | 1.64 | 1.64 | 3.28 | 1.64 |  | 3.28 | 1.64 | 6.56 | 50.82 | 0.996(0.004) |
|  | 8 | 59 | 5.17 | 3.45 |  |  | 5.17 | 10.34 | 3.45 |  |  | 6.9 |  |  | 1.72 | 3.45 |  | 60.34 | 0.990(0.007) |
|  | 9 | 66 | 7.58 | 6.06 |  | 1.52 | 3.03 | 6.06 | 6.06 | 4.55 |  | 1.52 | 3.03 | 3.03 | 1.52 | 1.52 | 3.03 | 51.52 | 0.995(0.004) |
|  | 10 | 70 | 4.29 | 4.29 | 1.43 |  | 1.43 | 17.14 | 1.43 | 1.43 | 2.86 |  | 4.29 |  | 1.43 |  | 2.86 | 57.14 | 0.996(0.003) |
|  | 11 | 56 | 3.57 | 1.79 |  |  | 5.36 | 16.07 | 3.57 |  | 1.79 |  |  |  | 7.14 | 3.57 | 3.57 | 53.57 | 0.997(0.004) |
|  | 12 | 23 |  | 8.7 | 4.35 | 17.39 |  | 17.39 | 4.35 |  | 13.04 | 4.35 | 4.35 |  |  |  |  | 26.09 | 0.996(0.014) |
|  | 13 | 67 | 10.45 | 1.49 |  | 2.99 | 1.49 | 13.43 |  |  | 5.97 | 2.99 |  |  | 4.48 | 2.99 | 2.99 | 50.75 | 0.996(0.004) |
|  | 14 | 16 | 6.25 | 6.25 |  |  |  | 12.5 |  |  |  |  |  |  |  |  | 18.75 | 56.25 | 0.983(0.028) |
|  | 15 | 26 | 3.45 | 17.24 |  | 3.45 | 10.34 | 3.45 | 3.45 |  | 3.45 |  |  |  |  | 3.45 | 3.45 | 48.28 | 0.993(0.011) |
|  | 16 | 54 |  | 1.85 |  | 3.7 | 3.7 | 9.26 | 3.7 | 3.7 | 5.56 | 5.56 |  |  | 3.7 |  | 5.56 | 53.7 | 0.996(0.004) |
|  | 17 | 82 | 6.1 | 1.22 |  |  |  | 23.17 |  | 1.22 |  |  | 6.1 | 1.22 | 1.22 | 4.88 | 4.88 | 50 | 0.995(0.003) |
|  | 18 | 42 | 11.9 | 7.14 |  | 4.76 |  | 9.52 | 7.14 |  | 2.38 | 4.76 |  |  |  | 2.38 | 4.76 | 45.24 | 0.995(0.006) |
|  | 19 | 56 |  | 6.9 |  |  | 3.45 | 3.45 | 3.45 | 10.34 |  | 3.45 |  |  |  |  | 6.9 | 62.07 | 1.000(0.010) |

Table S3. (continued)

|  |  |  | **Estimated percentage of mtDNA Haplogroup (%)** | | | | | | | | | | | | | | |  | Gene diversity (S.D.) |
| --- | --- | --- | --- | --- | --- | --- | --- | --- | --- | --- | --- | --- | --- | --- | --- | --- | --- | --- | --- |
| Group | Population | size | A | B4a | B5 | B5b | C | D* | D5 | F* | F1a1 | F1b | G2a | M7a | M8a | N* | N9a | others |  |
| Northern Minorities (NM) | 20 | 48 | 8.33 | 2.08 |  |  | 6.25 | 31.25 | 6.25 |  |  |  |  |  |  |  | 6.25 | 39.58 | 0.989(0.007) |
|  | 21 | 48 | 6.25 |  |  | 4.17 |  | 22.92 | 10.42 |  |  |  | 6.25 |  |  |  | 2.08 | 47.92 | 0.976(0.010) |
|  | 22 | 53 | 3.77 | 3.77 |  |  | 13.21 | 13.21 |  |  |  | 5.66 | 1.89 |  | 1.89 |  | 1.89 | 54.72 | 0.993(0.011) |
|  | 23 | 47 | 4.26 |  |  | 2.13 | 19.15 | 25.53 | 4.26 |  |  |  |  |  |  |  |  | 44.68 | 0.956(0.011) |
|  | 24 | 56 | 4.76 |  |  |  | 9.52 | 4.76 | 4.76 | 2.38 |  | 4.76 | 11.9 |  | 7.14 | 11.9 |  | 38.1 | 0.991(0.005) |
|  | 25 | 47 | 4.26 |  |  |  | 6.38 | 6.38 | 2.13 |  |  | 2.13 | 10.64 |  | 6.38 |  |  | 61.70 | 0.999(0.006) |
| Southern  Minorities (SM) | 26 | 56 | 12.5 | 1.79 | 1.79 |  | 1.79 | 12.5 | 1.79 | 7.14 |  | 3.57 |  |  |  |  |  | 57.14 | 0.996(0.004) |
|  | 27 | 96 | 3.13 | 5.21 |  | 3.13 | 9.38 | 12.5 | 3.13 | 8.33 |  | 3.13 |  |  |  | 1.04 | 4.17 | 46.88 | 0.993(0.003) |
|  | 28 | 395 | 0.76 | 10.13 | 0.25 | 0.51 | 2.28 | 0.51 | 0.76 | 1.27 | 2.78 | 2.53 |  |  | 0.25 | 0.25 | 3.04 | 74.68 | 0.987(0.002) |
|  | 29 | 142 | 4.93 | 7.04 |  | 0.7 | 7.04 | 2.82 | 2.11 | 2.11 | 2.82 | 2.11 |  |  | 1.41 | 0.7 | 2.11 | 64.08 | 0.983(0.005) |
|  | 30 | 38 | 5.26 | 7.89 |  |  | 10.53 | 5.26 | 5.26 | 2.63 |  |  |  |  |  |  |  | 63.16 | 0.994(0.008) |
|  | 31 | 55 | 5.45 | 1.82 |  |  |  | 14.55 | 1.82 | 3.64 |  | 3.64 |  | 1.82 |  | 3.64 |  | 63.64 | 0.997(0.004) |
|  | 32 | 83 | 2.41 | 1.2 |  | 1.2 | 4.82 | 1.2 | 4.82 | 2.41 |  |  |  |  | 3.61 | 1.2 | 2.41 | 74.7 | 0.992(0.004) |
