# Supplementary figures and images for: Mitochondrial DNA Evidence for a Diversified Origin of Workers Building Mausoleum for First Emperor of China
Source: PLoS One. 2008 Oct 1;3(10):e3275. doi: 10.1371/journal.pone.0003275 (PMC2557057; doi:10.1371/journal.pone.0003275)

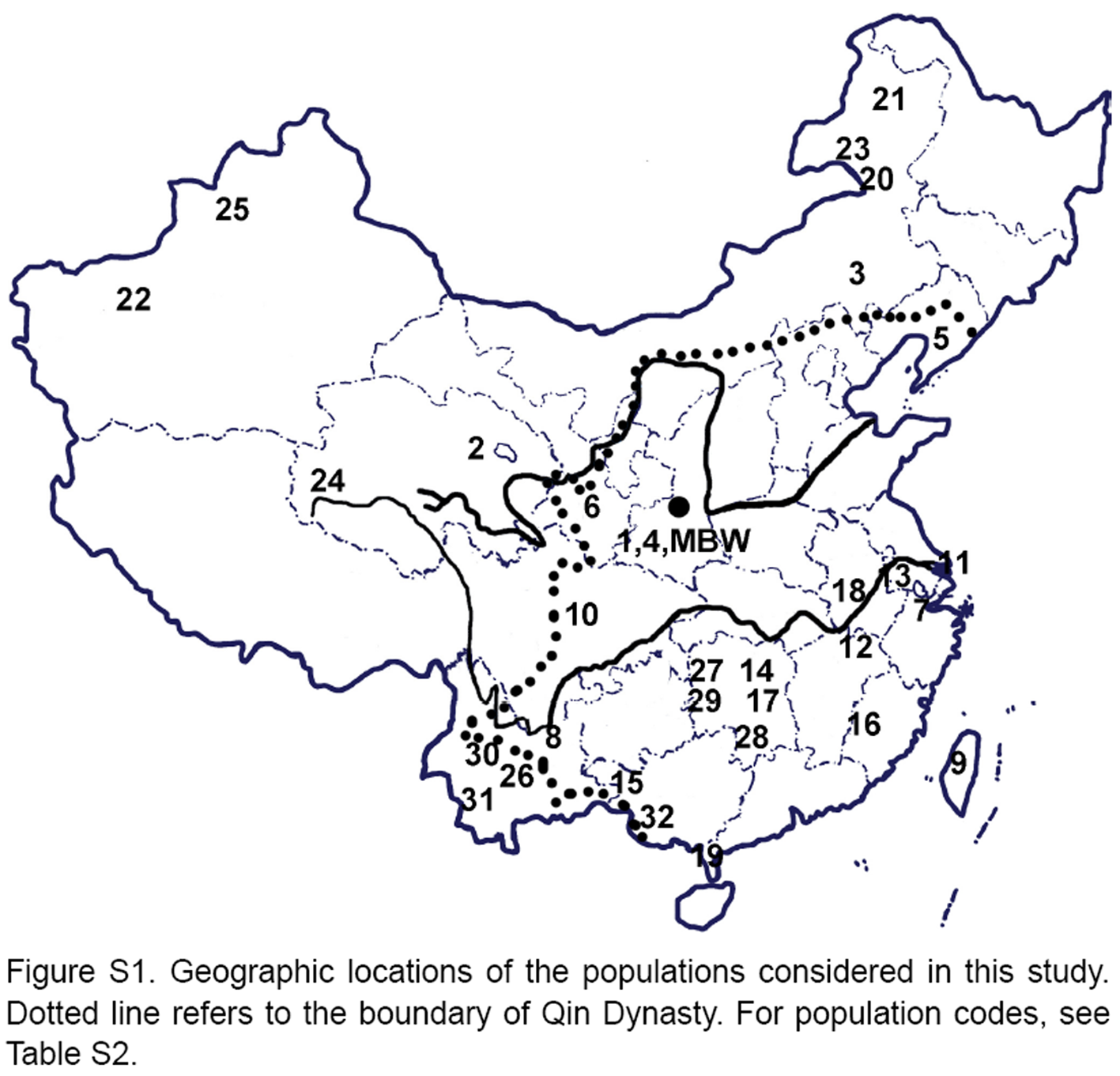

Supplement: Figure S1 — Geographic locations of the populations considered in this study. (6.74 MB TIF) [file pone.0003275.s004.tif]

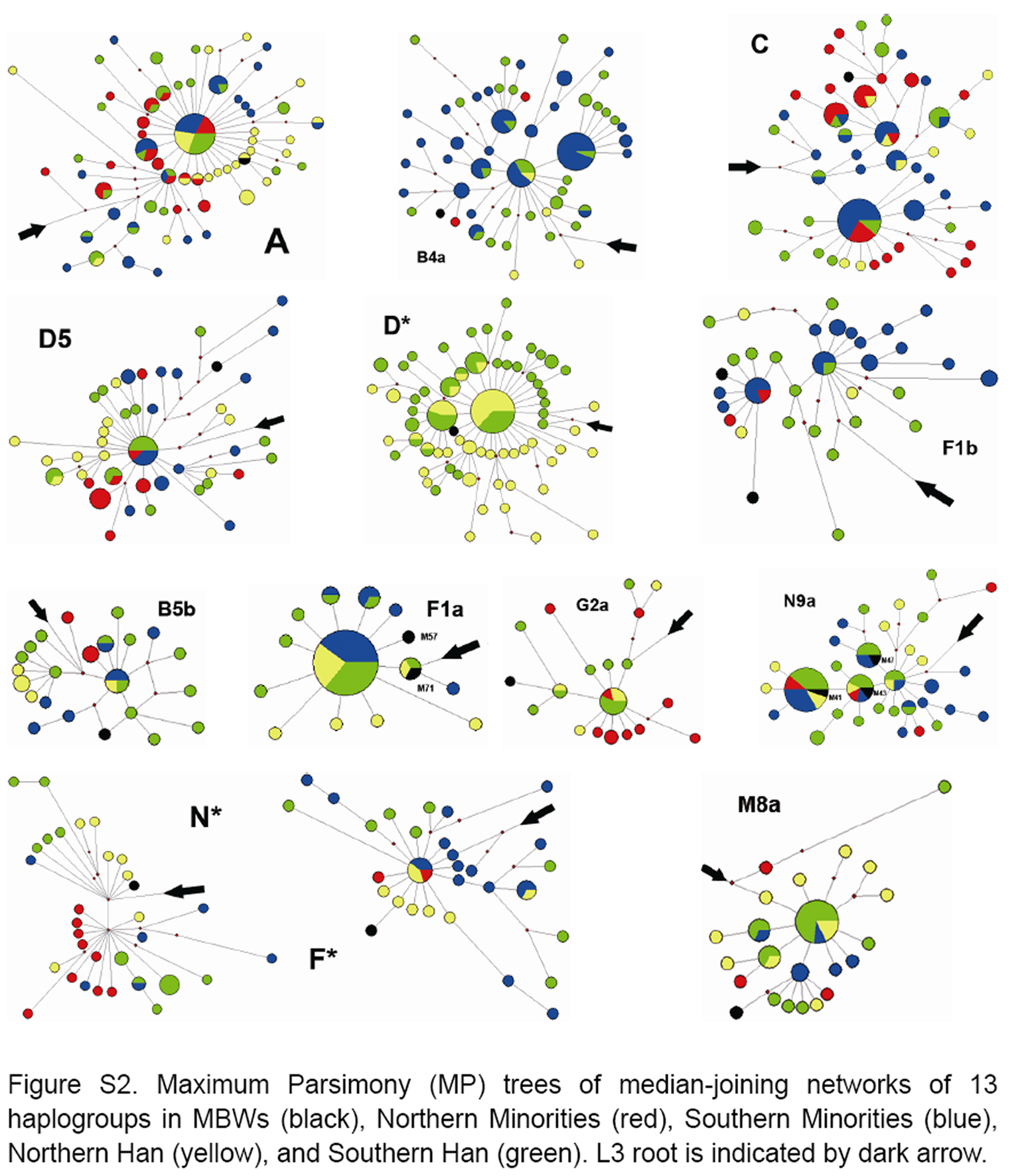

Supplement: Figure S2 — Maximum Parsimony (MP) trees of median-joining networks (9.69 MB TIF) [file pone.0003275.s005.tif]
